# Supplementary material for: Barcoded bulk QTL mapping reveals highly polygenic and epistatic architecture of complex traits in yeast
Source: eLife. 2022 Feb 11;11:e73983. doi: 10.7554/eLife.73983 (PMC8979589; doi:10.7554/eLife.73983)
Supplement: Transparent reporting form [file elife-73983-transrepform1.pdf]

Westbrook Centre
Milton Road
Cambridge CB4 1YG, UK

P 01223 855340
W elifesciences.org
T @elife

eLife's transparent reporting form

We encourage authors to provide detailed information *within their submission* to facilitate the interpretation and replication of experiments. Authors can upload supporting documentation to indicate the use of appropriate reporting guidelines for health-related research (see EQUATOR Network), life science research (see the BioSharing Information Resource), or the ARRIVE guidelines for reporting work involving animal research. Where applicable, authors should refer to any relevant reporting standards documents in this form.

If you have any questions, please consult our Journal Policies and/or contact us: editorial@elifesciences.org.

Sample-size estimation

- You should state whether an appropriate sample size was computed when the study was being designed
- You should state the statistical method of sample size computation and any required assumptions
- If no explicit power analysis was used, you should describe how you decided what sample (replicate) size (number) to use

Please outline where this information can be found within the submission (e.g., sections or figure legends), or explain why this information doesn't apply to your submission:

> The motivation for the size of the F1 panel (~100,000 strains) is outlined in the Introduction section of the main text. Appendix 3 provides simulation-based justification for this sample size.

Replicates

- You should report how often each experiment was performed
- You should include a definition of biological versus technical replication
- The data obtained should be provided and sufficient information should be provided to indicate the number of independent biological and/or technical replicates
- If you encountered any outliers, you should describe how these were handled
- Criteria for exclusion/inclusion of data should be clearly stated
- High-throughput sequence data should be uploaded before submission, with a private link for reviewers provided (these are available from both GEO and ArrayExpress)

Please outline where this information can be found within the submission (e.g., sections or figure legends), or explain why this information doesn't apply to your submission:

> Information on phenotyping assay replicates can be found on Appendix 2, and in the caption to Fig. 1E, and is summarized below.
>
> We created a single panel of ~100,000 strains. Phenotyping of the panel in each environment was performed in two independent replicate assays, each containing all ~100,000 strains. Phenotypic inference was either [i] performed on each replicate independently (for Fig. 1E and Fig.1-supp.4), or [ii] jointly across replicates (for all other analyses).

eLife Sciences Publications, Ltd is a limited liability non-profit non-stock corporation incorporated in the State of Delaware, USA, with company number 5030732, and is registered in the UK with company number FC030576 and branch number BR015634 at the address Westbrook Centre, Milton Road, Cambridge CB4 1YG.

Statistical reporting

- Statistical analysis methods should be described and justified
- Raw data should be presented in figures whenever informative to do so (typically when N per group is less than 10)
- For each experiment, you should identify the statistical tests used, exact values of N, definitions of center, methods of multiple test correction, and dispersion and precision measures (e.g., mean, median, SD, SEM, confidence intervals; and, for the major substantive results, a measure of effect size (e.g., Pearson's r, Cohen's d)
- Report exact p-values wherever possible alongside the summary statistics and 95% confidence intervals. These should be reported for all key questions and not only when the p-value is less than 0.05.

Please outline where this information can be found within the submission (e.g., sections or figure legends), or explain why this information doesn't apply to your submission:

Detailed statistical discussion of the QTL inference procedure can be found on Appendix 3.
All statistical tests reported in figures or text have enough explanation in their context, or will point to more detailed explanations in a different section.

(For large datasets, or papers with a very large number of statistical tests, you may upload a single table file with tests, Ns, etc., with reference to sections in the manuscript.)

Group allocation

- Indicate how samples were allocated into experimental groups (in the case of clinical studies, please specify allocation to treatment method); if randomization was used, please also state if restricted randomization was applied
- Indicate if masking was used during group allocation, data collection and/or data analysis

Please outline where this information can be found within the submission (e.g., sections or figure legends), or explain why this information doesn't apply to your submission:

QTL inference was performed on a *training* subset of the phenotyping and genotyping data (subset of strains), while inferred model performance was measured on the remaining *test* data. Data allocation into train or test was done at random.

Additional data files ("source data")

- We encourage you to upload relevant additional data files, such as numerical data that are represented as a graph in a figure, or as a summary table
- Where provided, these should be in the most useful format, and they can be uploaded as "Source data" files linked to a main figure or table
- Include model definition files including the full list of parameters used
- Include code used for data analysis (e.g., R, MatLab)
- Avoid stating that data files are "available upon request"

Please indicate the figures or tables for which source data files have been provided:

eLife Sciences Publications, Ltd is a limited liability non-profit non-stock corporation incorporated in the State of Delaware, USA, with company number 5030732, and is registered in the UK with company number FC030576 and branch number BR015634 at the address Westbrook Centre, Milton Road, Cambridge CB4 1YG.

Table 2 -- Source Data 1: tab2_data1.txt
Full results of GO analysis on pleiotropic genes.

Figure 1 - Source Data 1 : fig1_data1.txt
Genotyping coverage of all strains in our panel.

Figure 1 - Source Data 2 : fig1_data2.txt
Inferred genotype for resequenced clones in Chr. XVI window.

Figure 1 - Source Data 3 : fig1_data3.txt
Replicate fitness measurements in 30C.

Figure 3 - Source Data 1 : fig3_data1.txt
Phenotypic correlation across environments.

Figure 3 - Source Data 2 : fig3_data2.txt
Number of genes within each inferred QTL's confidence interval.

Figure 3 - Source Data 3 : fig3_data3.txt
Frequency of lead SNPs in 1,011 Yeast Genomes panel.

Figure 3 - Source Data 4 : fig3_data4.txt
Pairwise model similarity scores across environments.

Figure 4 - Source Data 1 : fig4_data1.txt
Degree and clustering coefficient of observed and simulated epistatic networks.

Figure 5 - Source Data 1 : fig5_data1.txt
Reconstruction fitness measurements and predictions from inferred models.

eLife Sciences Publications, Ltd is a limited liability non-profit non-stock corporation incorporated in the State of Delaware, USA, with company number 5030732, and is registered in the UK with company number FC030576 and branch number BR015634 at the address Westbrook Centre, Milton Road, Cambridge CB4 1YG.
